# The speciation and hybridization history of the genus *Salmonella*

**DOI:** 10.1099/mgen.0.000284

**Published:** 2019-07-26

**Authors:** Alexis Criscuolo, Sylvie Issenhuth-Jeanjean, Xavier Didelot, Kaisa Thorell, James Hale, Julian Parkhill, Nicholas R. Thomson, François-Xavier Weill, Daniel Falush, Sylvain Brisse

**Affiliations:** ^1^ Hub de Bioinformatique et Biostatistique – Département Biologie Computationnelle, Institut Pasteur, USR 3756 CNRS, Paris, France; ^2^ Institut Pasteur, Unité des Bactéries Pathogènes Entériques, World Health Organization Collaborating Centre for Reference and Research on * Salmonella *, Paris, France; ^3^ School of Life Sciences and Department of Statistics, University of Warwick, Coventry, UK; ^4^ Department of Microbiology and Immunology, University of Gothenburg, Gothenburg, Sweden; ^5^ Environmental Research Institute, University College Cork, Cork, Ireland; ^6^ Wellcome Trust Sanger Institute, Hinxton, UK; ^7^ The Milner Centre for Evolution, University of Bath, Bath, UK; ^8^ Institut Pasteur, Biodiversity and Epidemiology of Bacterial Pathogens, Paris, France

**Keywords:** *Salmonella*, speciation, hybridization, evolution, taxonomy, genomics

## Abstract

Bacteria and archaea make up most of natural diversity, but the mechanisms that underlie the origin and maintenance of prokaryotic species are poorly understood. We investigated the speciation history of the genus *Salmonella *, an ecologically diverse bacterial lineage, within which *S. enterica* subsp. *enterica* is responsible for important human food-borne infections. We performed a survey of diversity across a large reference collection using multilocus sequence typing, followed by genome sequencing of distinct lineages. We identified 11 distinct phylogroups, 3 of which were previously undescribed. Strains assigned to *S. enterica* subsp. *salamae* are polyphyletic, with two distinct lineages that we designate Salamae A and B. Strains of the subspecies *houtenae* are subdivided into two groups, Houtenae A and B, and are both related to Selander’s group VII. A phylogroup we designate VIII was previously unknown. A simple binary fission model of speciation cannot explain observed patterns of sequence diversity. In the recent past, there have been large-scale hybridization events involving an unsampled ancestral lineage and three distantly related lineages of the genus that have given rise to Houtenae A, Houtenae B and VII. We found no evidence for ongoing hybridization in the other eight lineages, but detected subtler signals of ancient recombination events. We are unable to fully resolve the speciation history of the genus, which might have involved additional speciation-by-hybridization or multi-way speciation events. Our results imply that traditional models of speciation by binary fission and divergence are not sufficient to account for *Salmonella* evolution.

## Data Summary

Illumina sequence data were submitted to the European Nucleotide Archive under project number PRJEB2099 and are available from INSDC (NCBI/ENA/DDBJ) under accession numbers ERS011101 to ERS011146. The multilocus sequence typing (MLST) sequence and profile data generated in this study were publicly available on the *Salmonella* MLST website from 2010 until the migration of the *Salmonella* MLST website to EnteroBase (https://enterobase.warwick.ac.uk/) and have been available from there since then.

**Impact Statement**

What is the family tree of *Salmonella *? To address this question, we first need to ask what a family tree is. The human family tree involves branching off from orangutans, gorillas, chimps and bonobos and then multiple species of hominids, including Neanderthals. The human family tree also includes hybridization events, including the recent genetic flow from Neanderthals into modern humans. In this paper we ask whether the *Salmonella* family tree is fully tree-like, with lineages splitting off sequentially from each other, or whether it in fact includes hybridization events. We explored this question by sampling the untapped diversity of *Salmonella* widely and by sequencing the complete genome of a representative sample of its lineages. We find that most of the time, species of *Salmonella* diverged vertically, but that there are some events involving rampant gene flow between distantly related lineages, which might be compared, for example, to the creation of a new species of apes by mixing the DNA of gibbons and gorillas. Our finding of long-distance hybridization poses a challenge for traditional bacterial taxonomy and for other approaches that assume that bacterial species trees can be summarized using binary splits.

## Introduction

Bacteria and archaea make up most of natural diversity, both in terms of species richness and biological functions [1, 2]. However, the mechanisms that underlie the origin and maintenance of prokaryotic species are poorly understood. It is often assumed that there is a single phylogenetic tree representing the relationships amongst prokaryotic taxa, with the branch lengths reflecting divergence times between them. However, bacteria and archaea acquire foreign DNA by homologous and non-homologous recombination and can recombine frequently, including in the genus *Salmonella* [3–10]. Within species, population structures can range from panmictic to highly clonal, depending on the recombination rate and the effective population size [11, 12]. High recombination rates can maintain genetic cohesion within a species, preventing divergence and speciation from occurring until barriers to gene flow develop. Recombination has been shown in laboratory experiments to be suppressed by nucleotide mismatches between donor and recipient [13, 14]. This property provides a potential mechanism for speciation. It has been shown by simulation that large effective population sizes and neutral genetic drift can precipitate speciation by increasing the average pairwise divergence between strains, leading to either binary or multi-way speciation events [5, 15].

Conversely, distinct new lineages or species can potentially arise almost instantaneously by hybridization of existing distantly related ones. Such large-scale hybridization events can occur at once by homologous recombination of large genomic regions (e.g. [16]), or through multiple exchanges of small chromosomal segments associated with ecological convergence [17, 18]. Therefore, to describe relationships between prokaryotes and understand patterns of species richness and phenotypic diversity, it is important to characterize the process of speciation and gene flow between species, including large-scale hybridization events [19, 20].

Large-scale hybridization events can be detected via lasting imprints in the genomes of the species in which they have occurred. For example, agricultural *Campylobacter coli* have been shown to be hybrids that have recently imported up to 23 % of their genome from *Campylobacter jejuni* [19, 20]. This leads to an intransitive (*i.e*. non-reciprocal) pattern of genetic relationships, such that agricultural *C. coli* lineages have high overall genetic similarity to non-agricultural *C. coli *, but this close relationship with each other does not imply a shared degree of genetic similarity to *C. jejuni *. Intransitivity is a particularly useful signal of hybridization events because it is likely to persist over evolutionary time even if genetic exchange has ceased.

Salmonellae are a prominent speciation model, where experimental and genomic studies of recombination and hybridization have been pioneered [4–10, 14]. The genus *Salmonella* is divided into a number of phylogroups, namely *bongori*, *enterica*, *salamae*, *arizonae*, *diarizonae*, *houtenae* and *indica* [21–23]. *Salmonella bongori* has been classified as a distinct species [23], while the other phylogroups are considered to be subspecies of a single species, *Salmonella enterica *. These taxa are further subdivided into serovars based on antigenic variation of flagellins and O-antigen.

Members of the genus *Salmonella* are major pathogens of humans and other warm-blooded animals. Human infections mostly involve *S. enterica* subspecies *enterica *, which can cause gastroenteritis, enteric fever and other infections [24, 25]. Other *S. enterica* subspecies, as well as the species *S. bongori *, are more typically isolated from cold-blooded animals or the environment, and are rarely reported from human infections [26].

Here we are concerned with evolutionary relationships rather than taxonomy and we designate phylogroups by names derived from these subspecies’ labels, e.g. Bongori, Arizonae, Diarizonae, etc., with Enterica representing subspecies *enterica*. We use italicized names such as *houtenae* to refer to previous subspecies designations, which sometimes differ from our phylogroup assignments. A seventh *S. enterica* subgroup (group VII) was distinguished based on multilocus enzyme electrophoresis and gene sequencing [27–29]. Note that the phylogenetic re-evaluation [30] of the proposed species *Salmonella subterranea* [31] shows that it does not belong to the genus *Salmonella *.

Phylogenetic analyses of the evolutionary relationships amongst the different *Salmonella* lineages have led to contradictory conclusions with several proposed phylogenetic trees [9, 28, 29, 32–42]. This lack of consensus might reflect technical issues with phylogenetic reconstruction, but a more biologically interesting possibility is that the history of *Salmonella* is not well characterized by a simple model in which speciation proceeds stepwise by irreversible binary fissions.

To test this hypothesis, we sampled the genetic diversity within the little studied groups from cold-blooded hosts and used whole-genome sequences from representative isolates of phylogroups to characterize the genetic relationships between them and to infer historical population splits and gene flow. We show that while a binary fission model of speciation works for some of the *Salmonella* lineages, there are several important historical events that cannot be characterized in this way.

## Methods

### Taxonomic sampling and multilocus sequence typing (MLST) analyses

A total of 367 strains (Table S1, available in the online version of this article) from outside the subspecies *enterica* were selected from the collection of the World Health Organization Collaborating Centre for Reference and Research on *Salmonella *, Institut Pasteur, Paris, France. This centre contains the reference strains of all *Salmonella* serovars and their variants. The 367 strains represented approximately one-third of currently described serovars outside subsp. *enterica* and they were selected to maximize the diversity of the antigenic formulae. MLST was performed on these strains using updated primers adapted from those of Kidgell *et al*. [43] to amplify DNA from *S. bongori* and all subspecies of *S. enterica *. The novel primers are described in Table S3; note that they were publicly available on the MLST website from 2008 until the migration of the *Salmonella* MLST website to EnteroBase and they have been available from there since then.

A phylogenetic tree (Fig. S1) was inferred from the median distance matrix of the seven genes with the algorithm BioNJ* [44]. The nucleotide diversity of groups was defined using the index π [45] with the program DnaSP [46] from the concatenation of the seven multiple sequence alignments (MSAs). Minimum spanning trees (Fig. S2) were built using the software tool BioNumerics (Applied-Maths, Belgium).

### Strain selection and genome sequencing

A set of 46 strains were selected for whole genome sequencing by Illumina 2×50 nt. The characteristics of the obtained *de novo* assemblies are summarized in Table S2. This set was completed with genome sequences gathered from the GenBank repository, leading to a total of 73 genomes (Table S2): *S. enterica* subsp. *enterica *, 16; subsp. *salamae*, 13; subsp. *arizonae*, 9; subsp. *diarizonae*, 10; subsp. *houtenae*, 6; subsp. *indica*, 4; *S. bongori *, 10; and VII, 2.

### Core gene construction and phylogenetic analysis

**Fig. 1.**
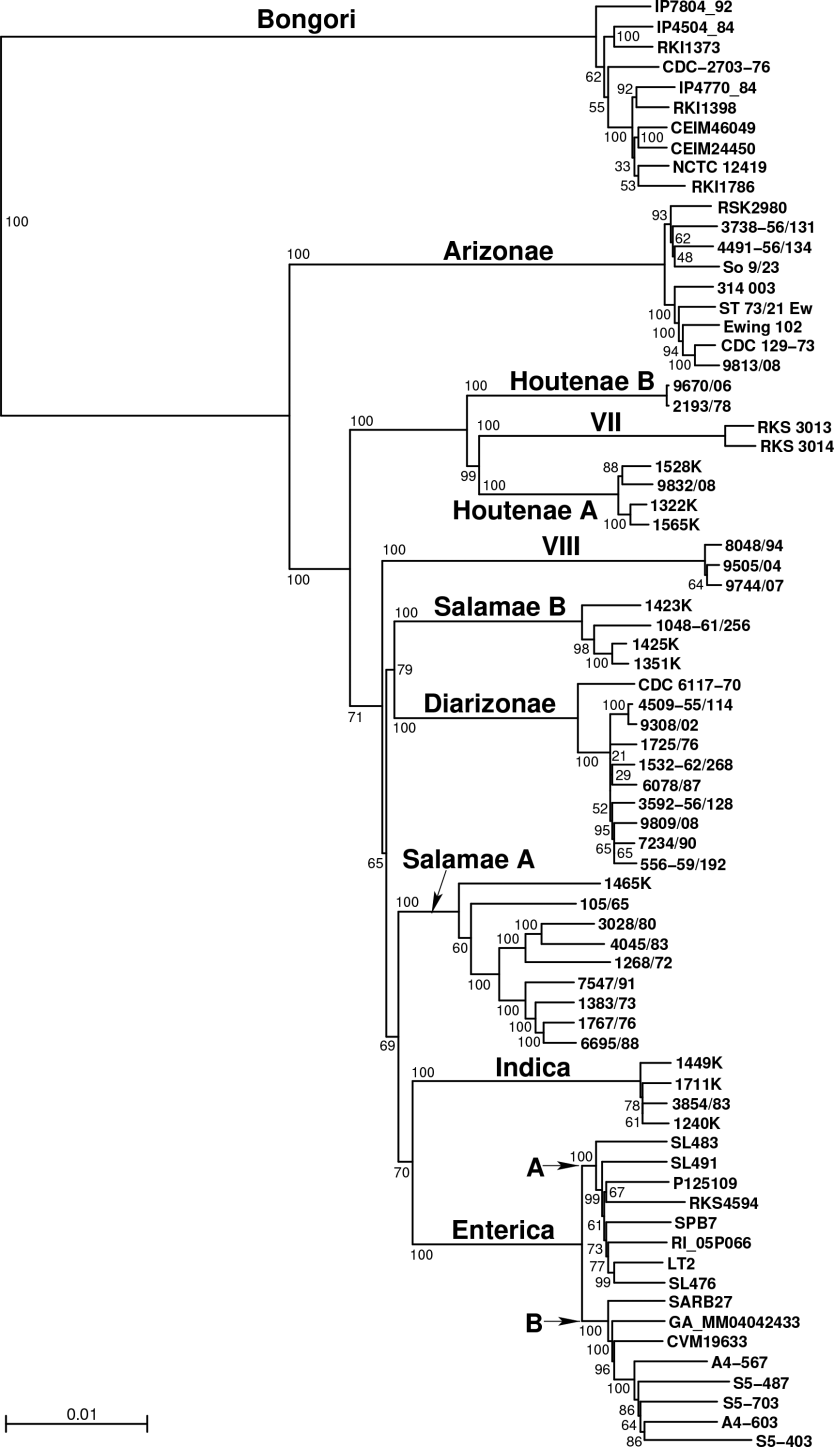
Phylogenetic tree of 73 *Salmonella* strains based on all shared core genes. The balanced minimum-evolution phylogenetic tree was constructed using FastME (see the Methods section). The 11 phylogroups are indicated above their ancestral branch; Enterica groups A and B are also indicated. Bootstrap-based branch support values are indicated next to the nodes. The scale bar corresponds to 0.01 nucleotide substitutions per character.

Each of the 4 423 protein sequences from *S. enterica* strain LT2 [47] was used as a query to perform blast similarity searches [48] against the genome sequence of each of the other 72 strains. Clusters of homologous sequences were built by considering only the first tblastn hit (*E*-value <10^−5^), and every cluster that did not contain 73 sequences (*i.e.* 1 per strain) was discarded. Next, orthology was assessed within each cluster by performing reciprocal tblastn, leading to 2 328 clusters of putative orthologous coding sequences from the core gene set of the 73 strains. For each of these clusters, sequences were translated, and an MSA was performed with ProbCons [49] and next back-translated to obtain a codon-level MSA. The 2 328 MSAs were concatenated into a supermatrix of 2 137 446 nucleotide characters that was used to infer a balanced minimum-evolution phylogenetic tree using FastME [50] from pairwise *p*-distances (Fig. 1). Branch support was assessed for each internal branch with an MSA-based bootstrap procedure with 1000 replicates. This procedure samples the MSA with replacement according to the same procedure as the standard bootstrap with nucleotide characters.

### Recombination analyses

**Fig. 2.**
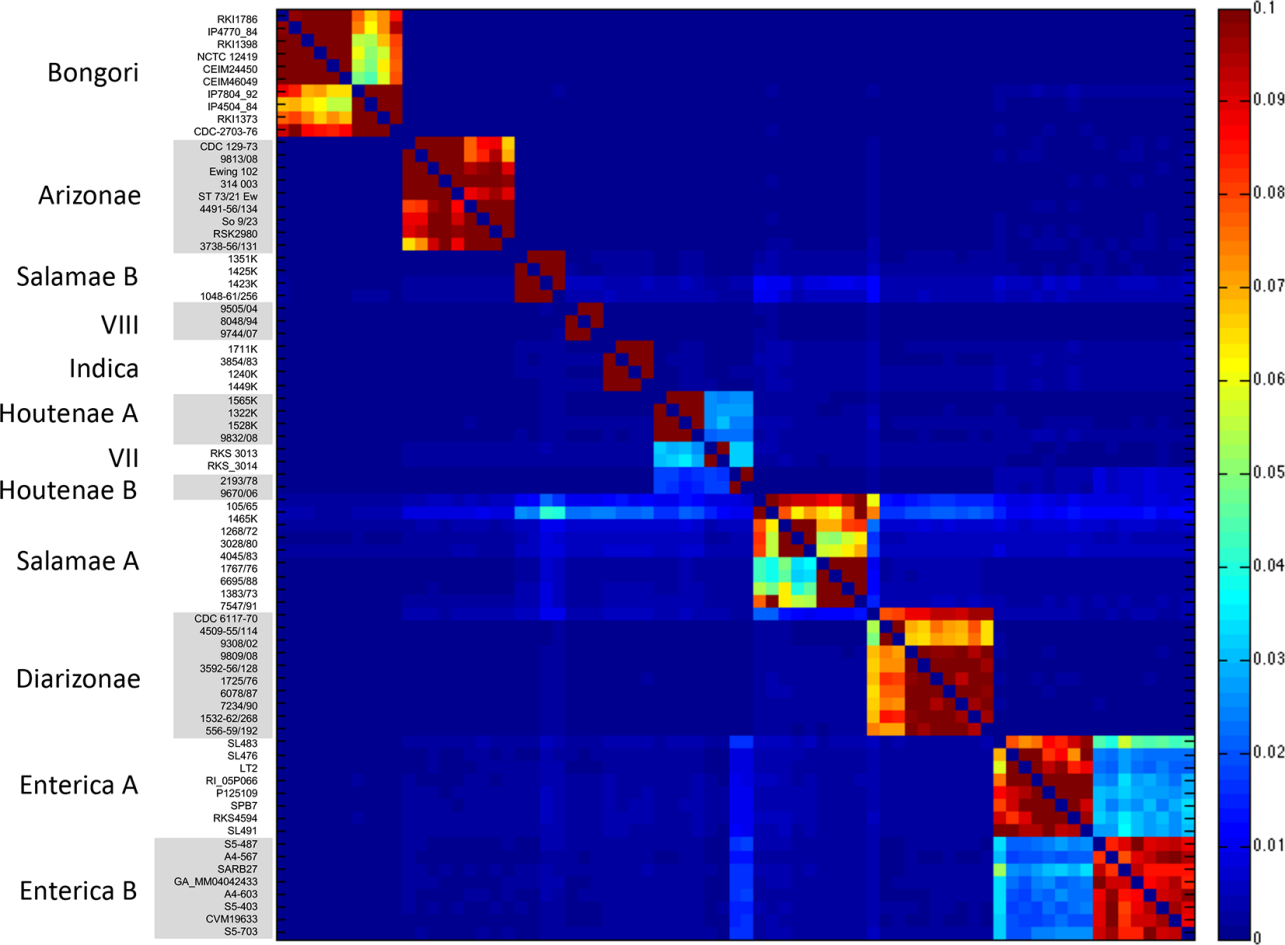
Coancestry matrix of 73 *Salmonella* genomes, computed using ChromoPainter. Each cell of the coancestry matrix is coloured according to the corresponding coancestry value (colour scale on the right), i.e. the amount of genetic material copied from a donor genome (columns) to a recipient genome (rows), with dark blue corresponding to 0 % and dark red corresponding to 10 %. The 73 strain names as well as the 12 phylogroups are indicated on the left.

**Fig. 3.**
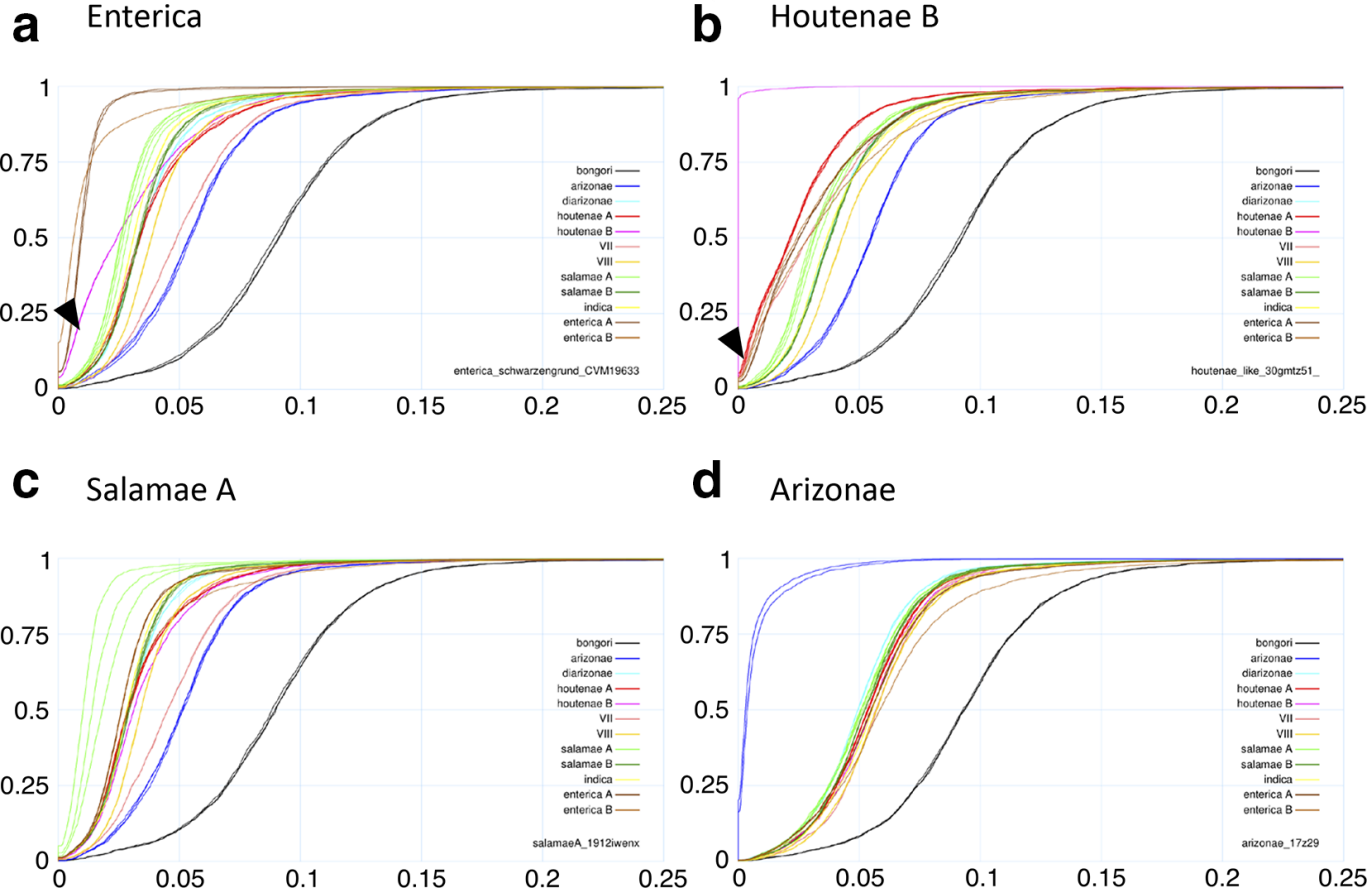
Cumulative curves of gene-by-gene distances between selected pairs of genomes. (a) Comparisons with Enterica (group B, serovar Schwartzengrund CVM19633). The arrowhead shows that 20 % (0.20, *y*-axis) of the genes of an Enterica B strain have less than 1 % (0.01, *x*-axis) divergence to Houtenae B. (b) Comparisons with Houtenae B (2193/78). The arrowhead shows that 5 % of the VII genome and 6 % of Houtenae A have less than 0.1 % divergence with Houtenae B. (c) Comparisons with Salamae A (1268/72). (d) Comparisons with Arizonae (CDC 129–73).

**Fig. 4.**
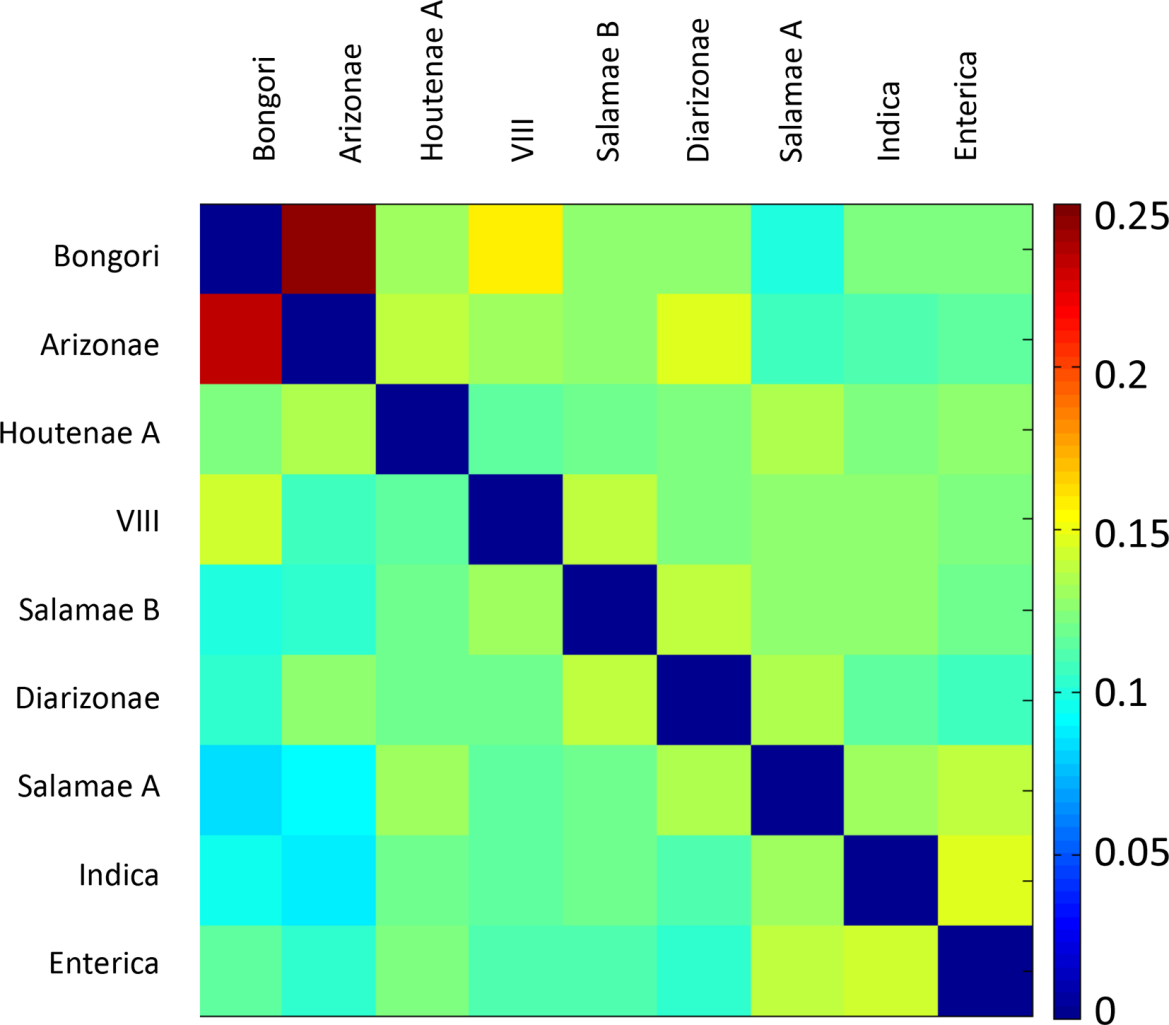
Coancestry matrix between nine unrelated genomes, computed using ChromoPainter. Each cell of the coancestry matrix is coloured according to the amount of genetic material copied from a donor genome (columns) to a recipient genome (rows).

**Fig. 5.**
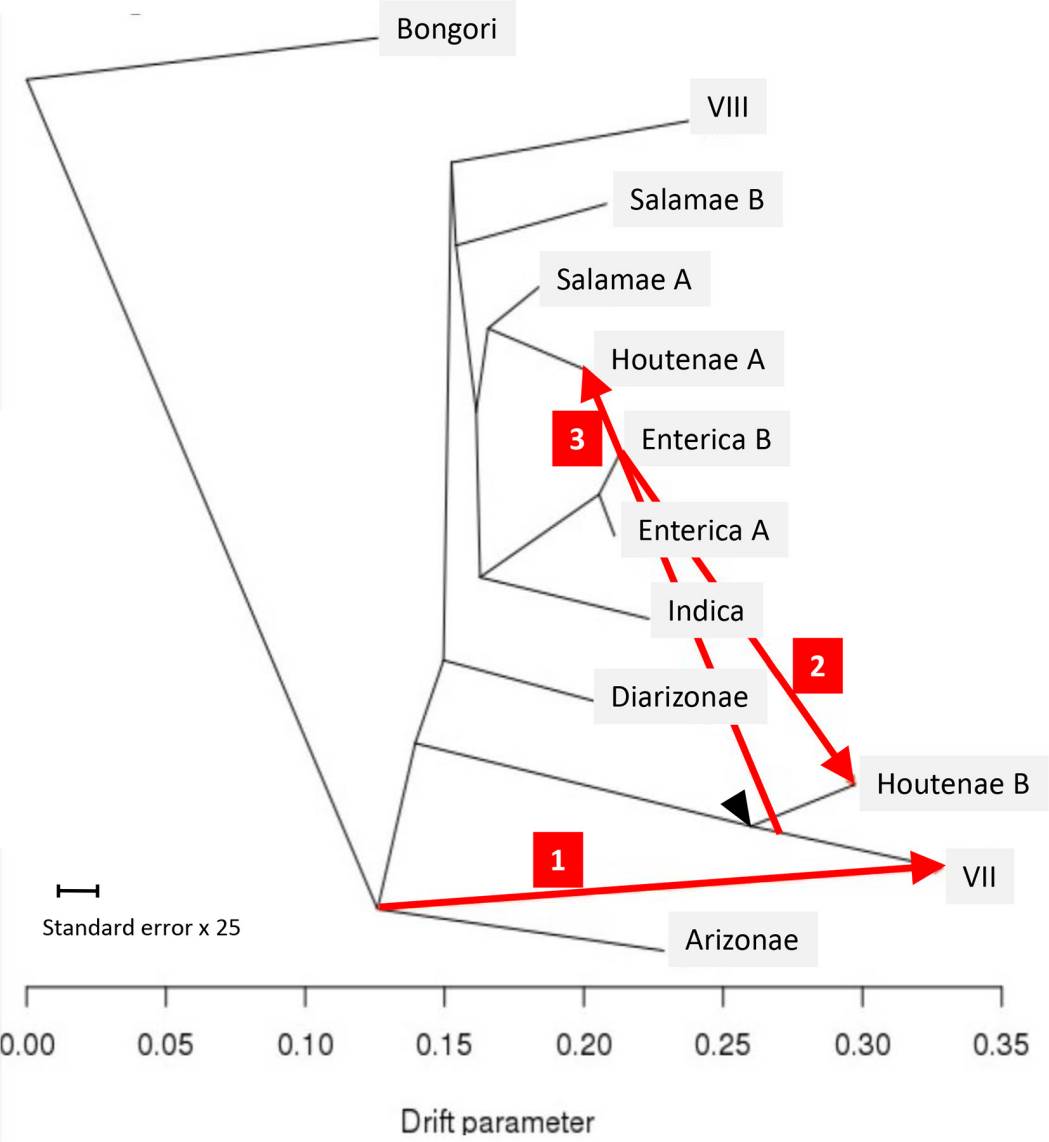
Treemix analysis of 12 genomes representative of phylogroup diversity. The black arrowhead indicates the position of the ancestor contributing to extant Houtenae A, Houtenae B and VII lineages. The red arrows indicate gene fluxes inferred by Treemix from one position on the tree to another.

We applied four separate and complementary methods to analyse the ancestral recombination events that occurred during the evolution of the genus *Salmonella *. Firstly, we applied chromosome painting to the 73 genomes, using ChromoPainter [51] to reconstruct each genome as a mosaic of all the others. The results were summarized as a heatmap of coancestry values, where each coancestry value corresponds to the number of fragments copied from one genome to another (Fig. 2). Secondly, we performed pairwise comparisons of genomes using a gene-by-gene approach. For each pair of genomes, we computed the genetic distances of all shared genes and the distribution of these distances was plotted as a cumulative curve (Fig. 3). Thirdly, the ChromoPainter analysis was repeated using only 9 unrelated genomes: 1 for each of the 12 phylogroups but excluding VII and Houtenae B due to recent shared ancestry with Houtenae A and considering Enterica A and B as a single group. Each genome was therefore reconstructed as a mosaic of the other eight unrelated genomes. This allowed us to explore deeper relationships between phylogroups; in contrast, if all genomes had been included, each genome from a phylogroup would copy mostly from other genomes of the same phylogroup (Fig. 2). The resulting coancestry matrix was plotted as a heatmap (Fig. 4). Fourthly, we applied the Treemix algorithm [52] with the parameter *K*=3 to 1 genome from each of the 12 phylogroups in order to reconstruct their relationships as a vertical phylogenetic tree augmented with horizontal transfer events (Fig. 5).

### Pan-genome analyses

Analysis of the accessory genome was performed using the Roary pan-genome pipeline version 3.6.2 [53]. Since the draft genomes were very unequally fragmented and the synteny information therefore was of variable reliability we used the ‘don't-split-paralogs’ option. The analysis was performed with a protein identity cut-off of 85 % and the core genome was defined as genes present in >99 % of the genomes studied. The Pearson correlations between the accessory gene content of the genomes were visualized using the R software corrplot package [54].

## Results and discussion

### *Salmonella* diversity

In order to survey the diversity of *Salmonella* outside *S. enterica* subsp. *enterica *, a total of 367 strains, comprising about a third of the known non-*e* *nterica* serovars, were selected from the World Health Organization Collaborating Centre for Reference and Research on *Salmonella* (Institut Pasteur, Paris, France) reference collection and subjected to MLST (Tables S1 and S2). A phylogenetic tree was inferred from MLST data (Fig. S1), revealing a novel group (labelled VIII) and suggesting a polyphyletic origin of *S. enterica* subsp. *salamae* (Salamae A and B) and of *S. enterica* subsp. *houtenae* (Houtenae A and B). The within-phylogroup nucleotide diversity (Fig. S1 inset) was highest in Arizonae (π=1.6 %) and lowest in Houtenae groups, Bongori, Salamae B and Diarizonae (π ranging from 0.35 to 0.42 %), while it was intermediate in Salamae A, Indica and Enterica. Minimum spanning tree analysis of the MLST profiles illustrates the genotypic diversity within each group (Fig. S2).

Based on MLST diversity, 46 genomes were chosen for genome sequencing and compared to 27 previously published genome sequences of Enterica, Arizonae and Bongori (Table S2). A phylogenetic tree based on the core gene set is shown in Fig. 1. This tree implies that *S. enterica* subsp. *salamae* is not a monophyletic group but instead forms two lineages with distinct evolutionary histories that we designate Salamae A and Salamae B. Whereas Salamae A contained 138 (88 %) of the *salamae* strains, Salamae B comprised 18 isolates collected from a human (1 isolate), a bat (1 isolate) or reptiles (16 isolates, including 6 from chameleons). In contrast, 49 (41.5 %) Salamae A isolates were from humans and only 34 (28.8 %) were from cold-blooded animals, suggesting important ecological and pathogenic differences between the two Salamae groups. *S. enterica* subsp. *houtenae* was also subdivided into two distinct phylogroups, here designated Houtenae A and Houtenae B, which clustered together with group VII on the tree. The genome-wide phylogenetic analysis also uncovered a hitherto unknown phylogroup, labelled VIII, made up of strains previously identified as either *S. enterica* subsp. *salamae *, *S. enterica* subsp. *diarizonae* or the former Hisingen serotype of *S. enterica* subsp. *enterica* [30]. The description of Salamae B, Houtenae B and VIII represents the first novel *Salmonella* phylogroups described since the identification of group VII by Selander and colleagues more than 25 years ago [29, 32]. Our analysis therefore defines 11 phylogroups within *Salmonella *. The phylogenetic tree in Fig. 1 also shows further subdivisions at shallower levels, including the division of *S. enterica* subsp. *enterica* into Enterica A and B, as previously described [5, 9]. Note that the genomes of the present study have been publicly available from the International Nucleotide Sequence Database Collaboration (INSDC) since 2011 and were used in a genome-based phylogenetic analysis of *Salmonella* by Alikhan *et al*. [55]; the three novel *Salmonella* groups were labelled therein as novel subspecies A (Houtenae B), B (VIII) and C (Salamae B) [55].

### Recent recombination between phylogroups

We used chromosome painting of the above set of 73 strains in order to investigate shared ancestry and recombination events between different phylogroups. Specifically, ChromoPainter uses a hidden Markov model to reconstruct each isolate as a mosaic of stretches of DNA of the other isolates in the sample [51]. Fig. S3 shows examples of the inferred mosaics. The results can be summarized as a heatmap indicating how many stretches from each sample are used in the reconstruction. The organism used in the reconstruction is assumed to be the most closely related for each stretch of DNA. Fig. 2 shows a heatmap illustrating the proportion of DNA used to paint each isolate across the genome, called the coancestry value. Each phylogroup shows higher coancestry values within the same phylogroup than it does with others. The highest coancestry value between strains in different phylogroups is between Houtenae A, Houtenae B and VII. However, Houtenae B shows higher Enterica ancestry (particularly with Enterica B) than Houtenae A or VII. The two deepest branching Salamae A strains show high levels of coancestry with several other groups, including Salamae B, Diarizonae, Indica and VIII. One strain of Enterica A (SL483) is exceptional in showing higher coancestry levels with Enterica B.

In order to test whether high coancestry between groups might be explained by recent recombination between them, we looked for evidence of sharing of very similar stretches of DNA between pairs of lineages [17] by plotting, for each pairwise comparison, the proportion of genes with divergence below a threshold increasing from 0 to 25 % (Fig. 3). Consistent with recent recombination between them, Enterica B and Houtenae B showed many more genes with very similar sequences than expected based on their position in the phylogenetic tree, with 20 % of the genes of an Enterica B strain having less than 1 % divergence to Houtenae B, compared to only 5 % between Enterica B and Houtenae A (Fig. 3a). These divergence curves are also consistent with recent recombination between Houtenae A, Houtenae B and VII. For example, approximately 5 % of the VII genome and 6 % of the Houtenae A one have less than 0.1 % divergence with Houtenae B (Fig. 3b), suggesting that there has been very recent recombination between these three phylogroups. There is no analogous signal of recent recombination between any of the strains of Salamae A or Salamae B with each other or with other phylogroups based on cumulative divergence curves (e.g. Fig. 3c). The smudged pattern of coancestry of the deeper branching Salamae A and Salamae B strains in Fig. 2 can potentially be explained by them retaining ancestral variants that have been lost by the rest of the phylogroup and therefore does not necessarily indicate recent recombination between lineages. Fig. 3d illustrates the absence of any signal of recent recombination with Arizonae.

### Evidence for hybridization in the origin of the phylogroups

We next examined the origins of the phylogroups themselves. Recombination events that predate the generation of the diversity observed within each phylogroup are unlikely to be picked up in the chromosome painting analysis (Fig. 2): members of a phylogroup that have inherited the same ancestrally imported stretch will be painted by each other for those stretches. Therefore, we selected a single strain from each phylogroup and performed a distinct chromosome painting analysis. We excluded VII and Houtenae B due to the recent shared ancestry with Houtenae A, and also included only a single representative for both Enterica A and Enterica B. The chromosome painting results (Fig. 4) show high coancestry between Bongori and Arizonae and between Indica and Enterica. These relationships can be interpreted using a vertical phylogenetic model, as they agree with a large number of different analyses, including ours (Fig. 1), that Arizonae is the earliest branching lineage within *S. enterica* and that Indica is a sister group of Enterica [9, 29, 38, 39].

On the other hand, the chromosome painting analysis revealed a large number of intransitive relationships (i.e. in which *x* has elevated coancestry with *y* and *y* has high coancestry with *z* but *z* does not have high coancestry with *x*). First, Diarizonae and Arizonae have high coancestry, as do Diarizonae and Salamae B, but Salamae B and Arizonae do not (Fig. 4). Second, Houtenae A and Salamae A have high coancestry with each other and the phylogenetic tree (Fig. 1) suggests that they are sister taxa. However, they have different relationships to other phylogroups. Houtenae A, but not Salamae A, shows high coancestry with Arizonae, while Salamae A shows higher shared ancestry with Indica and Enterica. Intransitive patterns of coancestry are also evident for the two taxa triplets VIII/Salamae B/Diarizonae and VIII/Salamae B/Bongori. An intransitive pattern is not predicted by any phylogenetic model and is indicative of mixture in the history. These observations suggest a complex pattern of homologous recombination events that predate diversification within phylogroups.

### A scenario involving three recent hybridization events

To complement the above results, we used Treemix to infer a history that allows for recombination events in the origins of the phylogroups. Treemix attempts to model the covariance matrix reflecting SNP sharing between strains by assuming a phylogenetic model of divergence via genetic drift, but with a limited number *K* of mixing events in the history. Our application of Treemix to *Salmonella* gave results that varied in important details, depending on the value of *K*. Each of the events that were identified at a given value of *K* had counterparts in the inference performed for higher values, but details of the inferred phylogenetic tree and the location and direction of the hybridization events were not consistent. For example, for *K*=1 and *K*=2 Houtenae A and Houtenae B are sister taxa whose common ancestor received genetic material from VII, while for *K*=3, VII and Houtenae B share a common ancestor, which contributed genetic material to Houtenae A.

We present the Treemix results for *K*=3 (Fig. 5) because all of the events inferred are supported by signals identified by chromosome painting and cumulative divergence (Figs 2–4). The Treemix results with *K*=3 imply that Houtenae A, Houtenae B and VII all have hybrid origins. All three of them received DNA from a shared lineage that branched between Arizonae and Diarizonae (black arrowhead, Fig. 5), but differ in the remaining source of their ancestry (red arrows, Fig. 5), which, according to the Treemix estimates, accounts for about half of their genome in all three cases, i.e. (i) ancestor of Arizonae to VII: 0.461; (ii) Enterica B to Houtenae B: 0.42; (iii) ancestor of VII to Houtenae A: 0.49. Note that according to this inference, no pure, or nearly pure, representative of this shared ancestral lineage is present in the sample, a feature that is likely to have contributed largely to the instability of the Treemix analysis and makes all types of evolutionary reconstruction considerably more challenging.

The second source for Houtenae B is inferred to be Enterica B (red arrow 2, Fig. 5), which is consistent with the results from chromosome painting and of the pairwise distances, as discussed above, and is consistent with recent genetic exchange having taken place. The second source for VII is inferred to branch at the same point as Arizonae in the tree. The deep position of this ancestry source is supported by the distribution of pairwise distances that VII has to shallower branching lineages such as Diarizonae or Salamae A, which are more similar to the distribution found for Arizonae than to that of either Houtenae A or Houtenae B (*e.g*. Fig. 3c). The distribution of distances of VII to Arizonae is similar to that of other shallow-branching lineages, suggesting that the recombination was not with Arizonae itself. Finally, the second source for Houtenae A branches next to Salamae A, which is consistent with the reconstructed position of Houtenae A in the phylogenetic tree in Fig. 1 and the high coancestry of Houtenae A and Salamae A in Fig. 4. However, unlike for Houtenae B, there is no signal of recent recombination of Houtenae A with other lineages in Fig. 2. Furthermore, the pairwise distance curves of Salamae A to Houtenae A and Houtenae B are comparable (Fig. 3c). These features imply that there has not been recent recombination between Houtenae A and Salamae A. Instead, they are consistent with the second source that contributed to Houtenae A being an unsampled sister taxa to Salamae A.

### Unequal evolutionary rates of the different taxa

One important feature of the phylogenetic tree (Fig. 1) is the different branch lengths leading to each phylogroup. This feature might be caused by either unequal substitution rates between lineages or by recombination, which can cause hybrid lineages to branch closer to the root. Evidence for unequal substitution rates comes, for example, from comparisons with Bongori or Arizonae, which can tentatively be treated as outgroups. Salamae A and Salamae B have smaller inter-phylogroup genetic distances than other lineages to either (Fig. 3d), despite the chromosome painting results indicating no evidence of elevated recombination between them. Furthermore, Salamae A and Salamae B show low genetic distances compared to potential sister lineages to all taxa, suggesting that they have substantially lower substitution rates than other groups. Because our reconstruction of *Salmonella *’s evolutionary history is incomplete and uncertain, we do not attempt to formally model the combined effect of lineage splitting, recombination and mutation on sequence diversity.

### Accessory genome relationships

Accessory genes contribute most to ecological specialization and the pattern of horizontal gene transfer among phylogroups might provide important complementary information regarding functional and ecological correlates of the recombination history that we inferred in this work [9]. We therefore analysed the pan-genome of the dataset, which with a protein identity cut-off of 85 % rendered a total pan-genome of 21 973 genes. Unfortunately, estimations of the strain relationships based on gene presence/absence and analysis of the shared ancestry revealed that the analyses were strongly affected by the fragmentation of the genomic assemblies (Table S2), as was particularly visible for the highly fragmented Diarizonae genomes (Fig. S4). Analysis of the horizontal gene transfer pattern among phylogroups therefore requires higher quality assemblies and will be the subject of future studies.

## Conclusions

We investigated the diversification and hybridization history within *Salmonella *, a group of prominent public health importance and an early model for microbial speciation and evolutionary studies. By sampling largely in the non-*enterica* subspecies, we uncovered three novel phylogenetic groups that had not been recognized since the last group, VII, was described in 1991. Our snapshot of diversity within phylogroups of *Salmonella* implies that recombination among phylogroups is relatively rare at any point in time, but that when it happens it can be with distantly related lineages rather than sister taxa and can involve large fractions of the core genome.

The three hybridization events that we have been able to elucidate with any degree of certainty are ongoing or took place in the recent past and all involved a lineage that is not present in unhybridized form in the dataset. This circumstance makes it challenging to estimate simple properties of the events, such as the direction of hybridization and the proportion of genome acquired from each source. We can nevertheless robustly conclude that the hybridization has involved at least three entirely different branches of the *Salmonella* tree and has led to the formation of three phylogroups, namely Houtenae A, Houtenae B and VII. Interestingly the latter group was inferred to be a ‘hybrid’ in early MLEE studies [29]. These observations suggest a question that is likely to be informative about the general nature of species boundaries in bacteria, namely what happened to make one lineage particularly prone to become involved in hybridization events?

We see less conclusive but nevertheless still strong evidence for hybridization events in the more distant past. Phylogenetic trees of *Salmonella* phylogroups are notoriously unstable, including in different analyses we have performed (data not shown). In particular, relationships amongst Salamae A, Salamae B, Diarizonae, Enterica and VIII are difficult to elucidate. The coancestry relationships between these lineages are highly intransitive (Fig. 4). One possibility is that this intransitivity is due to a complex multi-way speciation event [5], such that there is no true splitting order to infer. However, it may also represent hybridization events after stepwise speciation. The two lineages that branch deeply in the phylogenetic tree (Fig. 1), namely VIII and Diarizonae, both show evidence of shared ancestry with basal lineages, Bongori and Arizonae, respectively (Fig. 4), which is likely to have affected their branching position in the tree, which should therefore not necessarily be assumed to reflect the true evolutionary history.

The large-scale recombination events inferred in this work explain the difficulties in reconstructing the phylogeny of the genus that have led to multiple distinct hypotheses concerning the relationships among subspecies. The phylogenetic relationships that do appear to be reasonably certain are that Bongori split from the other phylogroups first, followed by Arizonae, and that Indica is the sister group to Enterica. Houtenae A seems to have been a sister taxon of Salamae A prior to its mixture events. These examples demonstrate that in the right circumstances, phylogenetic signal can be preserved over substantial time periods. Nevertheless, we have been unable to reconstruct a complete history of the genus *Salmonella* with any confidence.

In summary, our results demonstrate that bacterial species histories are complex. There is considerable phylogenetic signal in the data, consistent with the evolution and long-term persistence barriers to gene flow between lineages, but also examples for hybridization events that may reverse species boundaries, sometimes between taxa separated by large genetic distances, rather than between sister taxa. These results mean that phylogenetic trees displaying relationships between species will often represent considerable simplifications of evolutionary history and in the worst case they can be entirely misleading. At present, the frequency of hybridization and complex speciation events is unknown, as evolutionary history has only been investigated in this way in a very small number of taxa. Further work in multiple taxa will establish how common these events are and elucidate the evolutionary and ecological factors that precipitate them.

## Data bibliography

Criscuolo, A, Thomson, N. R. and Brisse, S. European Nucleotide Archive. PRJEB2099. (2010).

## Supplementary Data

Supplementary File 1

Supplementary File 2
