# Supplementary material for: The speciation and hybridization history of the genus Salmonella
Source: Microb Genom. 2019 Jul 26;5(8):e000284. doi: 10.1099/mgen.0.000284 (PMC6755497; doi:10.1099/mgen.0.000284)

Supplementary Figures

Figure S1. BioNJ* tree of 382 *Salmonella* strains based on seven housekeeping gene sequences. The scale bar corresponds to 0.01 nucleotide substitutions per character. Black circles indicate the 73 strains used for subsequent genome-based analyses. The inset shows the nucleotide diversity index π (black dots) and its standard deviation (horizontal bars) estimated from the concatenation of the seven genes for each phylogroup (Houtenae comprises Houtenae A and Houtenae B).

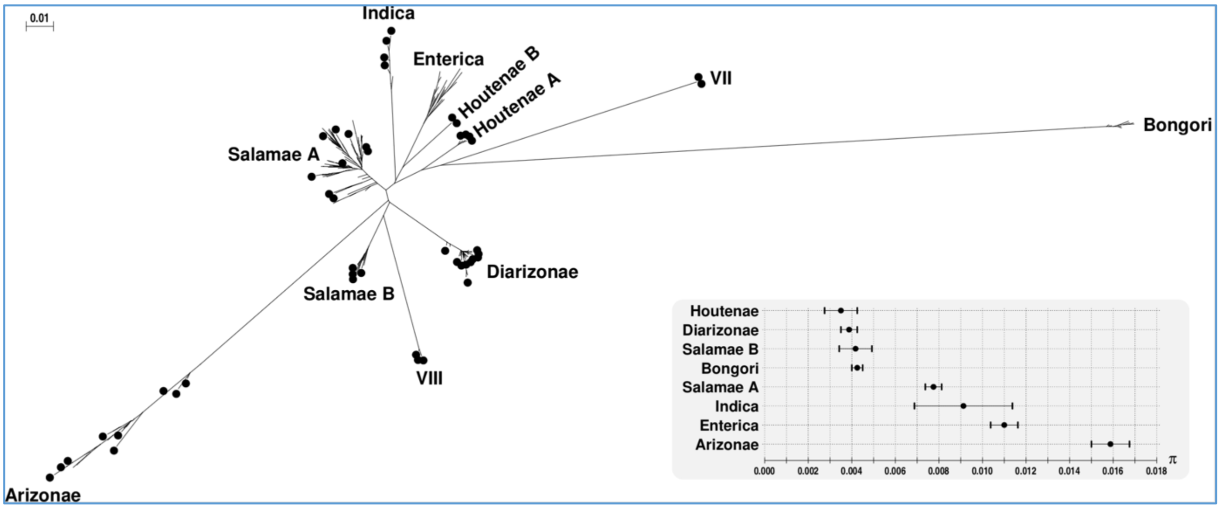

Figure S2. Minimum spanning tree representations of the genotypic diversity within *Salmonella* groups. The minimum spanning trees were constructed for each group based on number of mismatches among MLST allelic profiles. Strains selected for genome sequencing are represented by blue sectors (or blue circles when only one strain shared the corresponding genotype). Grey zones surround groups of sequence types that are connected successively by single allelic mismatches and are equivalent to clonal complexes or 'eBURST' groups (Achtman *et al*., 2012).

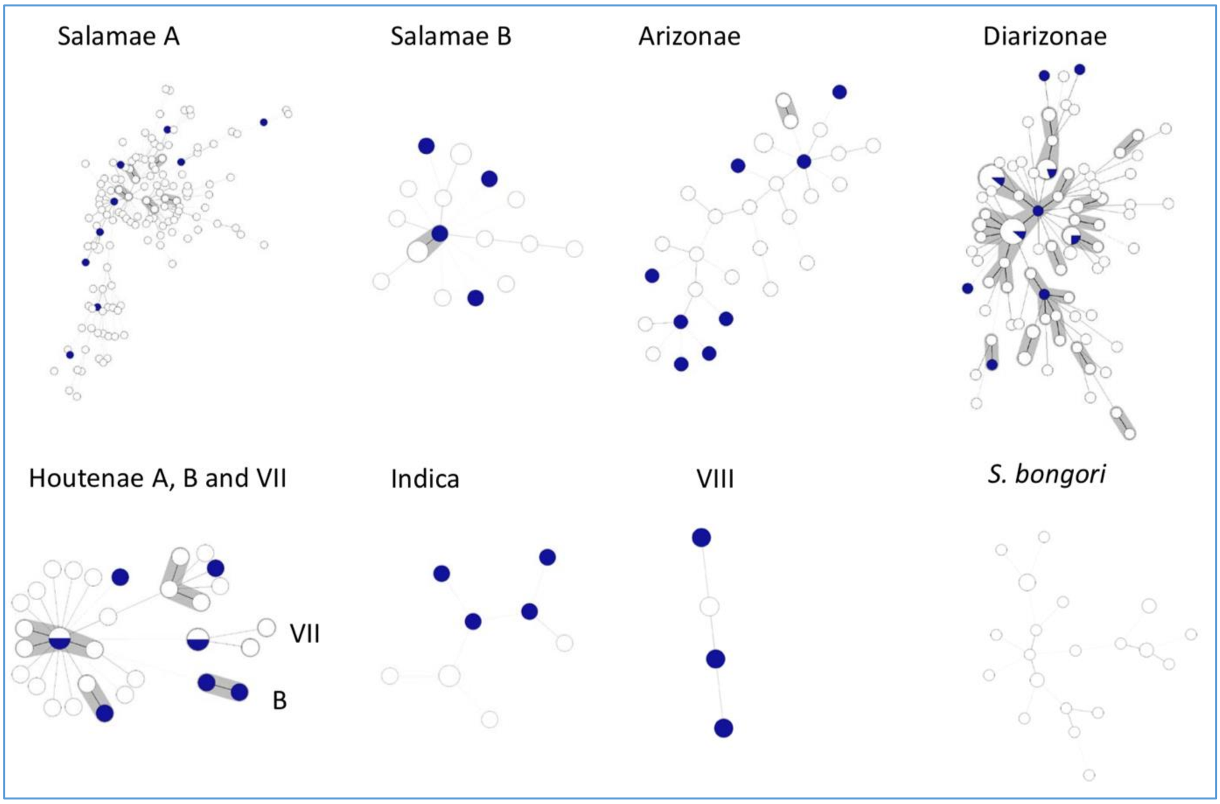

Figure S3. Illustrative examples of CHROMOPAINTER mosaics. A unique color is associated with each population inferred by FineStructure (top part). The genomic positions in the first 50 genes of 5 selected genomes are colored with a mixture of colors which represents the probability of copying from each of the populations. Bold black vertical lines represent the limits between genes which are shown side by side. Coloring happens most of the time with the same or similar color as the population to which the genome belongs, with a few short exceptions which represent recent recombination events.

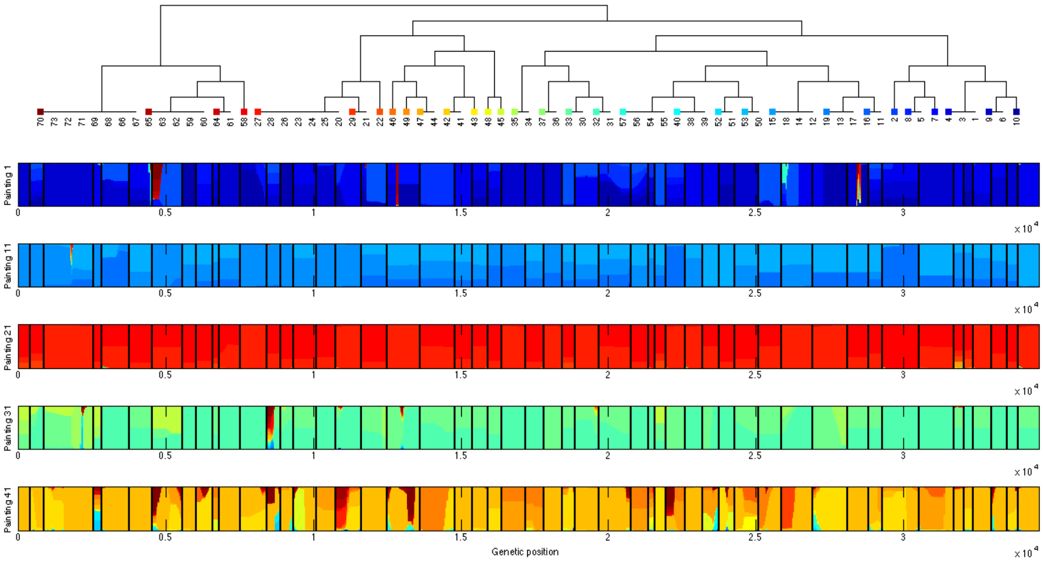

Figure S4. Heatmap of the proportion of shared genes

Strains are ordered according to the phylogeny in Figure 1 (left). The proportion of shared genes was computed from the ROARY output with a protein identity cut-off of 85% and the "don't split paralogs" option.

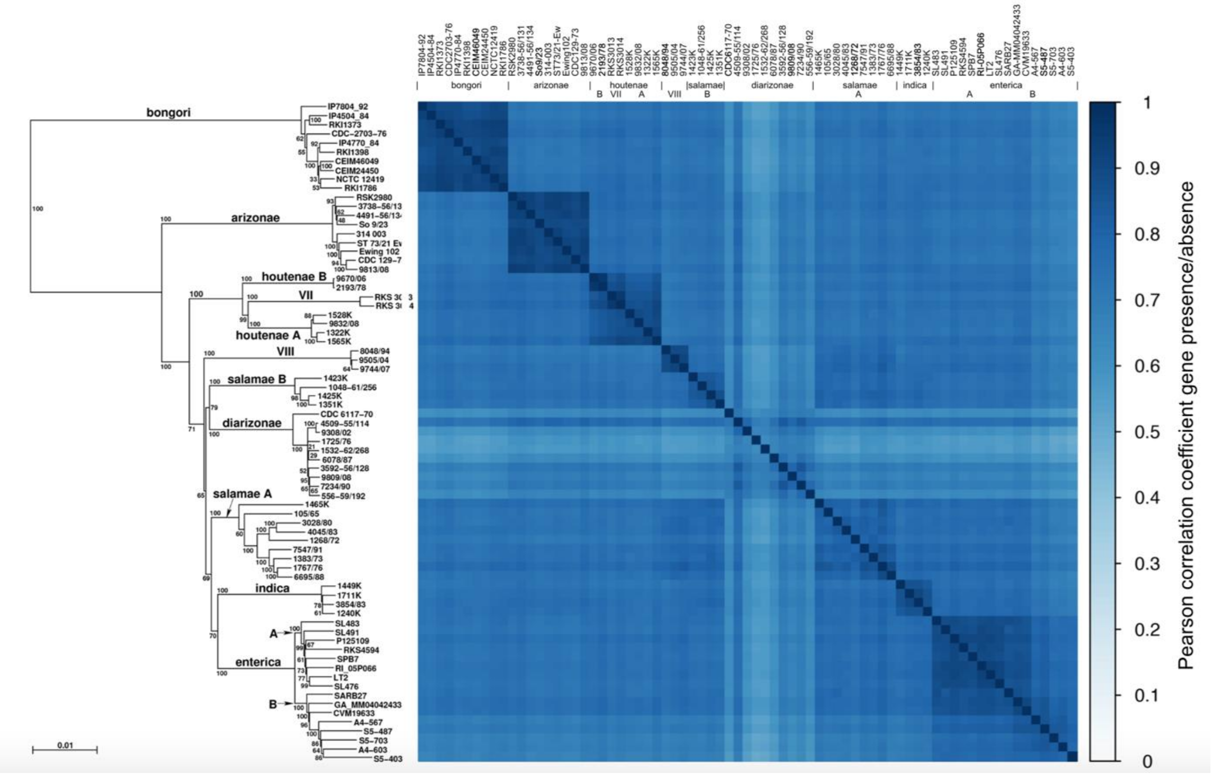

Supplement: Supplementary File 1 [file mgen-5-284-s001.pdf]
